# Profiling learning approaches among Saudi postgraduate medical trainees: advancing education in the Vision 2030 era

**DOI:** 10.3389/fmed.2025.1654690

**Published:** 2025-09-01

**Authors:** Reem S. AlOmar, Nouf A. AlShamlan

**Affiliations:** Department of Family and Community Medicine, College of Medicine, Imam Abdulrahman Bin Faisal University, Dammam, Saudi Arabia

**Keywords:** medical education, learning approaches, postgraduate clinical trainees, health policy, vision 2030

## Abstract

**Introduction:**

Understanding learning approaches among postgraduate clinical trainees is essential for shaping educational policies that align with Saudi Arabia’s Vision 2030 and its healthcare transformation agenda. This study examines the predominant learning approaches among Saudi postgraduate trainees and explores their implications for medical education reform and workforce development policies.

**Methods:**

A nationwide cross-sectional online survey was administered to 2,010 trainees using the Approaches and Study Skills Inventory for Students (ASSIST). Bivariate and multinomial regression analyses were conducted to assess associations between learning approaches and trainee characteristics.

**Results:**

A total of 1,187 trainees responded (response rate: 59.05%). The most common learning approaches were strategic (39.98%) and deep (37.22%). Female trainees were more likely to adopt the deep approach (adjusted RRR = 1.51, 95% CI: 1.02–2.24), while a history of failing a promotion exam significantly predicted the adoption of a surface learning approach (adjusted RRR = 2.85, 95% CI: 1.44–5.61).

**Discussion:**

Learning approaches among postgraduate trainees varied by sex and academic experience, with policy-relevant implications. These findings highlight the need for personalized, competency-based educational frameworks that account for diverse learner profiles. Tailored pedagogical strategies, informed by these patterns, can enhance workforce preparedness, support equitable training opportunities, and inform healthcare education policy reforms aligned with Vision 2030.

## 1 Introduction

Medical trainees are expected to possess a higher degree of autonomy and exhibit an initiative to learn during their training process (1). This is true especially for postgraduate clinical trainees, where the learning climate is a combination of the physical and psychological environment as well as the overall teaching and learning conditions. For clinical trainees, a positive environment is crucial for effective learning in different rotations and models of clinical and workplace-based learning (2).

In the Kingdom of Saudi Arabia (KSA), postgraduate clinical training programs are monitored by the Saudi Commission for Health Specialties (SCFHS). The SCFHS had endorsed the Canadian Medical Education Directives for Specialists framework, commonly known as CanMEDS for all medical training programs it oversees (3). Furthermore, and in light of the health sector transformation program put forth under the Vision 2030 in the KSA, several initiatives to improve clinical training programs were introduced, and these include but are not limited to redesigning several programs, training of trainers, planning educational solutions and stress-coming skills support (4, 5).

Learning approaches may be defined as the intentions and motivations that are in part formed by the trainees” responses to certain situations (6). Understanding the predominant learning approaches among trainees is critical since learning approaches are essential in planning, designing, and implementing effective learning and teaching strategies (7). Previous studies have found that trainees can embrace different learning strategies depending upon the subject they are studying and have also found that learning processes and strategies affect the learning outcomes in terms of what is remembered and learned (7, 8).

The Approaches and Study Skills Inventory (ASSIST) is commonly used to determine a participant’s learning approach. This 18-item questionnaire tool, once scored, divides learning approaches into three main types, namely, deep, surface, and strategic. With the deep approach, trainees are able to correlate previous knowledge learned with the new, as well as being able to study in a more comprehensive manner, thereby retaining information for longer periods of time. In contrast, surface learners tend to choose the most rapid way to accomplish a specific task, do not show interest nor ask in-depth questions of the subject being taught, and memorize it without fully understanding it. Whereas with strategic learners, they are organized learners and effective time managers who attempt to gain the highest outcome by focusing on the most important objectives and tasks (1, 9).

Several international studies have explored learning approaches among health professions trainees using the ASSIST tool, revealing consistent patterns across diverse settings. For example, studies from Singapore and Sri Lanka reported that postgraduate trainees predominantly adopted the strategic approach (10, 11). These findings underscore the global relevance of understanding learning approaches to inform educational strategies and support learner success.

Only one study has used the ASSIST tool in the KSA, focusing on undergraduate medical students, and found that 40.59% of all students were deep learners (1). Notably, the study also observed that students’ tendency to adopt a certain learning approach varied by their level of study. This observation, along with the absence of research specifically targeting postgraduate clinical trainees, prompted this study to explore their predominant learning approaches and assess whether associations exist between these approaches and sociodemographic or academic characteristics. In addition to describing these patterns, the study aims to inform medical educators and program directors about how learning approaches may guide tailored teaching strategies, with policy-relevant implications that align with ongoing educational enhancement efforts under Saudi Arabia’s Vision 2030.

## 2 Materials and methods

### 2.1 Study design and participants

This cross-sectional study invited postgraduate clinical trainees from all SCFHS affiliated residency programs within the KSA to participate during February 2024. Undergraduate medical students were excluded.

### 2.2 Ethical considerations

The institutional review board of Imam Abdulrahman Bin University had approved the study with reference number (IRB-2024-01-087). The participation in this study was voluntary. Consent to participate in the study was obtained prior to answering the questions. No personally identifiable information was asked. The study complied with the principles of the Declarations of Helsinki.

### 2.3 Sample size and sampling techniques

The minimum required sample size was 1,013. This was based on a total population of postgraduate trainees in the KSA that was 63,434 according to the statistical yearbook of the Ministry of Health (12). An expected frequency of 40.59% was used in the calculation which was from a previous study of medical students that had belonged to the deep approach, at 95% CI with a margin of error of 3% (1). The sample size was calculated via the Epi Info software V.5.5.2.

Postgraduate clinical trainees of both sexes were invited to participate. A non-probability sampling technique was used where several data collectors were assigned to overview the data collection process. This was achieved by sending a link which contained the survey to trainees registered phone numbers. To avoid duplication of responses, the link did not allow for any multiple responses.

### 2.4 Data collection tool

The data was collected by means of an online-based, self-administered questionnaire. The questionnaire covered both sociodemographic characteristics such as age (mean ± SD) and sex (males, females). Also, academic and training specific characteristics were collected, these included whether the participant had previously failed during the undergraduate medical school (yes, no), and the type of the current postgraduate training program (medical, e.g., pediatrics, emergency medicine, dermatology, surgical, e.g., plastic surgery, obstetrics and gynecology, urology or others such as a master’s degree), the year of study (years 1–2, years 2–3, year 5 and above), whether the participant had failed during a promotion exam (yes, no).

The ASSIST short version tool was used which when scored was used as the main outcome of the study. The ASSIST tool consists of 18 questions measured on a Likert scale ranging from 1 to 5 (1 = disagree, 2 = somewhat disagree, 3 = unsure, 4 = somewhat agree, 5 = agree). The responses allow the investigators to identify the three learning approaches of participants where the deep learning approach includes items 2, 6, 10, 12, 15 and 17. The strategic approach includes items 3, 5, 7, 9, 11 and 13. The surface approach includes 1, 4, 8, 14, 16 and 18. The tool is scored by summing the responses to those specific items to a new variable, and the highest score to one of those approaches meant that it was the predominant approach to that participant. Participants who had scored equally to two approaches are removed from the analysis (1, 11). The reliability score for the ASSIST questionnaire was 0.87 indicating good internal consistency.

### 2.5 Statistical analysis

The data was analyzed in STATA software version 15 (13). Variables were described as either means ± SD after checking for normality or by frequencies and proportions. Bivariate associations were performed through a series of independent samples t-tests and *ꭓ*^2^ tests where appropriate. Unadjusted and adjusted multinominal logistic regression analyses were used to compute the relative risk ratios (RRRs) along with their 95% confidence intervals (CIs) while using the strategic approach as the base outcome. The level of significance was set to less than 0.05. Model diagnostics were performed to ensure good model fit.

## 3 Results

### 3.1 Sociodemographic and educational characteristics of postgraduate trainees

A total of 2,010 had accessed the online questionnaire, of those 1,187 had responded, which gave a response rate of 59.05%. However, 279 were excluded from the analysis due to having equal scores to two learning approaches. Of the remaining 908 trainees, the average age was 27 years ± 2.79 SD. Also, 55.29% were females compared to 44.71% males. Over 75% had not previously failed medical school. With regards to the type of postgraduate program, 60.13% were medical, 24.78% surgical and only 15.09% were enrolled in other types of postgraduate training such as a master’s program. Over half the participants were in their first year of training (54.74%), and only 13.95% had failed a promoting exam (Table 1).

**Table 1:** Sociodemographic and educational characteristics of postgraduate trainees.

Characteristics	*N* (%) 908 (100.00)
Age (mean ± SD)	27.19 (02.79)
Sex	
Males	406 (44.71)
Females	502 (55.29)
Previously failed during medical school	
Yes	209 (23.02)
No	699 (76.98)
Type of postgraduate program	
Medical	546 (60.13)
Surgical	225 (24.78)
Others (Such as a masters)	137 (15.09)
Year of study	
Year 1–2	497 (54.74)
Year 3–4	272 (29.96)
Year 5 or more	139 (15.30)
Ever failed during a promotion exam^¥^	
Yes	83 (13.95)
No	512 (86.05)

^¥^Computations are based on only 595 trainees, as 313 were in year one and are yet to be examined.

### 3.2 Sociodemographic and educational characteristics of postgraduate trainees according to the three learning approaches

Table 2 describes the sociodemographic and educational characteristics of postgraduate trainees according to the three learning approaches. Most trainees were found to adopt the strategic approach followed by the deep approach (39.98 and 37.22% respectively). No statistically significant difference was observed for age or sex. However, a higher proportion of males adopted the strategic approach, whereas females preferred the deep approach (41.63 and 40.04% respectively). A statistically significant association was found between previously failing during medical school and the learning approach, where trainees with no history of failure during medical school mostly adopted the strategic approach (41.20%). With regards to the type of postgraduate program, medical trainees equally adopted the deep and strategic approach (40.11%), whereas for surgical trainees, a clear preference for the strategic approach was observed (37.78%) (*p* = 0.03). As for previously failing a postgraduate promotion exam, of the total 595 trainees, those who had failed mostly adopted the surface approach whereas those who had not failed mostly adopted the strategic approach (43.37 and 42.19% respectively) (*p* < 0.001).

**Table 2:** Sociodemographic and educational characteristics of postgraduate trainees according to the three learning approaches.

Characteristics	Learning approaches *N* (%)		
	Deep approach 338 (37.22)	Strategic approach 363 (39.98)	Surface approach 207 (22.80)
Age (mean ± SD)	27.34 (02.94)	27.21 (2.84)	26.89 (02.38)
*p*-value	0.21		
Sex			
Males	137 (33.74)	169 (41.63)	100 (24.63)
Females	201 (40.04)	194 (38.65)	107 (21.31)
*p*-value	0.13		
Previously failed during medical school			
Yes	69 (33.01)	75 (35.89)	65 (31.10)
No	269 (38.48)	288 (41.20)	142 (20.32)
*p*-value	0.004		
Type of postgraduate program			
Medical	219 (40.11)	219 (40.11)	108 (19.78)
Surgical	73 (32.44)	85 (37.78)	67 (29.78)
Others (Such as a masters)	46 (33.57)	59 (43.07)	32 (23.36)
*p*-value	0.03		
Ever failed during a promotion exam^¥^			
Yes	23 (27.71)	24 (28.92)	36 (43.37)
No	191 (37.30)	216 (42.19)	105 (20.51)
*p*-value	<0.001		

^¥^Computations are based on only 595 trainees, as 313 were in year one and are yet to be examined.

### 3.3 Multinominal logistic regression of the three learning approaches

The multinominal logistic regression analyses found that age was not a significant predictor for the predominant learning approaches (Table 3). With regards to the deep approach in comparison to the strategic approach, females were observed to be more likely to adopt it (Adjusted RRR = 1.51, 95% CI = 1.02–2.24). According to the unadjusted model, trainees who had previously failed during medical school were observed to be more likely to adopt the surface approach in learning in comparison to the strategic approach (Unadjusted RRR = 1.73, 95% CI = 1.08–2.77), however this was not significant after adjustment. Previously failing a promotion exam was a significant predictor for adopting the surface approach more than the strategic approach, both before and after adjustment (Adjusted RRR = 2.85, 95% CI = 1.44–5.61).

**Table 3:** Multinominal logistic regression of the three learning approaches.

Predictors	Deep approach^*^				Surface approach^*^			
	Unadjusted RRR	95% CI	Adjusted RRR	95% CI	Unadjusted RRR	95% CI	Adjusted RRR	95%CI
Age	1.02	0.95–1.08	1.03	0.96–1.10	0.91	0.84–0.99	0.94	0.87–1.02
Sex								
Males	*Ref*							
Females	1.46	1.01–2.13	1.51	1.02–2.24	0.93	0.61–1.42	0.97	0.61–1.52
Ever failed in medical school								
No	*Ref*							
Yes	0.98	0.62–1.54	0.99	0.59–1.63	1.73	1.08–2.77	1.24	0.71–2.17
Postgraduate program								
Medical	1.43	0.86–2.35	1.51	0.88–2.58	1.31	0.72–2.38	1.48	0.78–2.83
Surgical	1.19	0.67–2.13	1.38	0.74–2.58	2.07	1.09–3.94	2.00	0.97–4.09
Other (Masters)	*Ref*							
Ever failed in a promotion exam								
No	*Ref*							
Yes	1.08	0.59–1.98	1.17	0.58–2.38	3.08	1.75–5.43	2.85	1.44–5.61

*Strategic approach is the reference outcome category. RRR, Relative risk ratio.

### 3.4 Sex differences in the adoption of the learning approaches according to the type of postgraduate program

**Figure 1:**
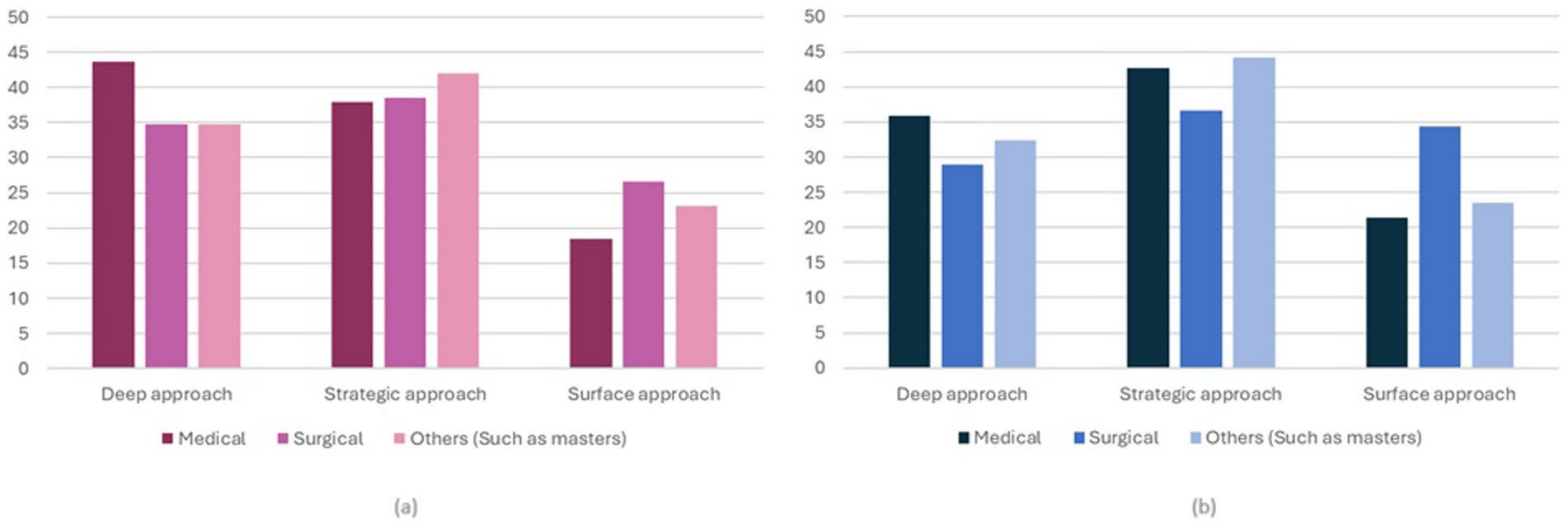
Percentage of adoption of learning approaches according to the type of postgraduate program by sex **(a)** Females; **(b)** Males.

Figures 1a,b shows females’ and males’ adoption of learning approaches according to the type of postgraduate program. For females, medical trainees were seen to prefer the deep approach and least prefer the surface approach, whereas for surgical trainees, they were seen to prefer the strategic approach and least prefer the surface approach. Trainees enrolled in other types of programs such as a master’s program were also seen to prefer the strategic approach (Figure 1a).

For males, and unlike females, medical trainees were observed to prefer the strategic approach in learning. Surgical trainees were seen to mostly adopt the strategic approach, followed by the surface approach, whereas for other types of programs, the strategic approach was also preferred (Figure 1b).

## 4 Discussion

This study aimed to identify the predominant learning approaches among postgraduate clinical trainees in the KSA and to provide policy-relevant insights for medical educators to support national strategies such as Saudi Arabia’s Vision 2030. Medical education for postgraduate trainees is critical as it bridges foundational medical knowledge with advanced clinical practice. Understanding and accommodating these preferences can enhance learning effectiveness, engagement, and ultimately, patient care outcomes. Trainees’ preferred learning approaches often vary according to their specialties, reflecting the unique demands and skillsets of each field. To our knowledge, in the medical educational field, no previous literature has targeted this group of individuals with most studies focusing on undergraduate medical students. Due to the very strict entry criteria to medical school, undergraduates are considered a highly homogenous group in terms of characteristics and also in their approach to learning (1, 14). However, with postgraduate clinical trainees, where graduates pursue highly different fields of study varying in the type of the program itself, either medical, surgical, or purely research, or in the years of study which varies between 3 to 7 years of study, it becomes important to understand their predominant learning approaches in order to seize the current climate of medical education improvement and set recommendations for higher success.

### 4.1 Sociodemographic and educational characteristics of postgraduate trainees according to the three learning approaches

In this study, the strategic approach was the most common approach, followed by the deep approach. This contrasts with the only other Saudi research which utilized the ASSIST tool among undergraduates in which the deep approach was the most common (1). However, internationally, two previous studies, one in Singapore and the other in Sri Lanka, had found similar results to the current study in which the predominant learning approach was the strategic closely followed by the deep approach (10, 11). These were also among postgraduates. Postgraduate trainees may feel pressured to prioritize job completion over in-depth understanding due to heavy workloads, prolonged shifts, and patient care duties (15). This pressure could cause trainees to adopt a strategic learning approach where they prioritize completing tasks right away over fully interacting with the course material.

The current findings also show that trainees with no history of failure during medical school mostly adopted the strategic approach. On the other hand, trainees who had previously failed during medical school were observed to be more likely to adopt the surface approach to learning in comparison to the strategic approach. However, adjustment analyses revealed that this difference was not statistically significant. Similarly, a previous failure in a postgraduate promotion exam was a significant predictor of adopting the surface approach more than the strategic approach, whereas those who had not failed mostly adopted the strategic approach. In agreement with results from the current study, a study among pre-clinical undergraduate students in Dominica showed a significant association between learning approaches and academic performance of students. When compared to low-achieving students, high-achieving students expressed a preference for strategic and deep learning approaches. Additionally, low achievers reported using the surface approach to learning most of the time (16). One more study using a sample of occupational therapy students in Norway found a correlation between greater exam scores and lower surface approach scores and higher strategic approach scores (17). A comparable and very recent study with 1,010 Egyptian nursing students by Dogham et al. revealed a significant association between surface learning approaches and academic stress. Elevated levels of academic stress can have a negative impact on students’ motivation and involvement in the learning process. This can result in feelings of overwhelm, lack of interest, or burnout, which may cause students to adopt a surface learning strategy (15).

In contrast to the studies that identified sex as a significant factor influencing students’ learning approaches, no statistically significant difference was observed in the present study (15, 16). However, a higher proportion of males adopted the strategic approach, whereas females preferred the deep approach. A similar observation was reported in a Saudi study among undergraduate medical students (1). This observation was also seen in other parts of the world. For example, in Egypt and in India females were also more likely to adopt the deep approach when compared to males (18). Understanding the disparities in approaches to learning between males and females may facilitate postgraduate programs in providing sex-specific resources and support. For instance, offering focused seminars or counseling services that address female and male trainees’ unique learning needs may help to alleviate such differences (15). Upon further analysis of the sex-based differences between the postgraduate clinical trainees’ programs and the different learning approaches, our results highlight the complex interactions between sex and specialty-related variables that influence the learning approaches of postgraduate trainees. By tailoring educational strategies to align with trainees’ specialty-specific needs and learning approaches, medical educators can foster deeper knowledge retention, improve clinical performance, and support the development of well-rounded, competent physicians who are better prepared to meet the complex challenges of modern healthcare systems.

### 4.2 Implications for medical educators in Kingdom of Saudi Arabia in light of vision 2030

The findings of this study offer critical insights for medical educators in the KSA, particularly within the framework of Vision 2030. The predominance of deep and strategic learning approaches among postgraduate trainees underscores the necessity for educational strategies that foster critical thinking, self-directed learning, and reflective practice. Vision 2030 emphasizes the transformation of the healthcare sector through workforce development and the enhancement of medical education programs to align with international standards. This includes a shift toward competency-based education, which necessitates curricula that support diverse learning approaches and cater to the individual needs of trainees (5).

The observed variations in learning approaches based on sex, specialty, and academic experience highlight the importance of personalized educational interventions. For instance, the higher prevalence of deep learning approaches among female trainees and those in surgical specialties suggests that tailored pedagogical methods could enhance learning outcomes across different demographics. Furthermore, targeted educational strategies, including mentorship, reflective practice modules, and leadership development initiatives tailored to female trainees, may not only enhance individual academic outcomes but also contribute to broader systemic goals of health sector advancement under Vision 2030. This aligns with the health sector transformation program of improving the quality of healthcare education and ensuring that all healthcare professionals are equipped with the necessary competencies to meet the evolving needs of the population (19).

Moreover, the strategic learning tendencies among trainees who are involved in research-based training programs suggest that integrating further research opportunities and teaching responsibilities into postgraduate programs could promote more effective learning strategies. This approach supports a culture of research and innovation within the healthcare sector (19).

### 4.3 Limitations of the study

The study does have some limitations. Firstly, the findings’ generalisability may be impacted by the use of a non-probability sampling technique. Second, participant subjectivity may have an influence on study findings. Moreover, the academic performance of trainees may be impacted by other factors such as individual variances, mitigating circumstances and learning motives that were not taken into account during this study. Other limitations include the cross-sectional design, which cannot establish the causality of the associations, and the reliance on self-reported data, which may have introduced biases.

## 5 Conclusion

This is the first study to apply the ASSIST tool to postgraduate clinical trainees in the KSA in order to understand the different learning approaches adopted by them. The strategic approach was most commonly used by Saudi trainee’s whereas the surface approach was the least used approach. The study had identified that sex was a statistically significant predictor in adopting a certain approach, where females were observed to highly adopt the deep approach than males in comparison to the strategic approach. Furthermore, having previously failed a postgraduate promotion exam was a significant predictor for adopting the surface approach compared to the strategic approach. Given that sex was observed to be a factor, the study recommends the use of sex-specific training resources such as seminars or educational counseling to promote a better learning environment.

## Data Availability

The raw data supporting the conclusions of this article will be made available by the authors, without undue reservation.
